# 

**Published:** 1904-09-15

**Authors:** 


### CONTENTS.

The Preparation of Enamel Walls and Margins for Fillings, - - *By L. P. Bethel, M.D., D.D.S.* 433
Office Management, - *By Henry C. Raymond, D.D.S.* 453
Regulation of Fees, - - *By Perry F. Hines, D.D.S.* 462
Mat Gold and How to Use it, *By J. L. Young, D.D.S.* 471
EDITORIAL, - - - - - - - - 476
ANNOUNCEMENTS, - - - - - - - - 479
BIBLIOGRAPHY, - - - - - - - - 480
INDENTS, - - - - - - - - - 482

